# An enhanced iTransformer-based early warning system for predicting automotive rental contract breaches

**DOI:** 10.1371/journal.pone.0319786

**Published:** 2025-03-20

**Authors:** Ming Jiang, Dongpeng Peng, Haihan Yu, Shu Chen

**Affiliations:** School of Internet Economics and Business, Fujian University of Technology, Fuzhou, China; LUMSA: Libera Universita Maria Santissima Assunta, ITALY

## Abstract

Economic losses in the car rental industry due to customer breaches remain a critical issue. The rapid growth of the vehicle leasing market has given rise to a pressing concern for enterprises, namely the economic loss, vehicle idleness, and service quality degradation that are often associated with customer default. This study proposes an innovative vehicle rental early warning system that incorporates the improved DBSCAN clustering technique and the iTransformer model. The enhanced DBSCAN technique, which employs a snow ablation optimizer (SAO) algorithm, establishes an electronic barrier and integrates the iTransformer model for trajectory prediction. This enables the real-time monitoring of potential customer defaults and the reduction of economic losses that leasing companies may incur as a result of customer defaults. The system identifies and prevents default risks in a timely manner through a comprehensive analysis of vehicle driving data, thereby safeguarding the interests of corporate entities. The system employs vehicle driving data provided by a Chinese company to accurately identify the vehicle’s resident location and predict future trajectory, effectively preventing customer defaults. The experimental results demonstrate that the model is highly effective in predicting the vehicle’s resident location and future trajectory. The mean square error (MSE), mean absolute error (MAE), and location error reached 0.001, 0.003, and 0.08 kilometers, respectively, which substantiates the model’s efficiency and accuracy. This study has the additional benefit of providing effective warnings to customers of potential default behavior, thereby reducing the economic losses incurred by enterprises. Such an approach not only ensures financial security but also enhances operational efficiency within the industry. Furthermore, it offers robust support for the sustainable development of the car rental industry.

## 1. Introduction

With the rapid development of China’s market economy and the rise in residents’ income levels, significant changes have occurred in consumption structures and concepts. Car leasing, as an emerging service, has provided users with personalized, flexible, and convenient travel options, while also offering social benefits such as resource conservation, pollution reduction, and traffic congestion alleviation [1]. Since China first ventured into the car rental business in 1989, the industry has experienced rapid growth, bolstered by strong policy support [2]. However, this rapid expansion has also unveiled numerous challenges, particularly the frequent occurrence of customer default behaviors, which include late payments, overdue return of vehicles, unauthorized borrowing of vehicles, and boundary violations such as vehicles entering restricted areas (e.g., border zones, vehicle dismantling sites). However, this rapid expansion has also unveiled numerous challenges, particularly the frequent occurrence of customer defaults. These defaults not only cause economic losses to enterprises but also adversely affect service quality and industry reputation.

Customer default behaviors in car rental primarily include failure to pay rent on time, overdue return of vehicles, unauthorized borrowing of vehicles, and boundary violations. Boundary violations refer to situations where rental vehicles enter restricted or dangerous areas, such as border zones and vehicle dismantling sites. Customer default behaviors in car rental primarily include failure to pay rent on time, overdue return of vehicles, and unauthorized borrowing of vehicles. These actions increase operational costs for enterprises and pose risks such as vehicle damage and loss. The economic instability during the pandemic has exacerbated these issues, with more customers unable to pay rent on time, leading to a higher incidence of contract breaches. Additionally, car rental contract breaches defaults can lead to legal disputes, further increasing legal costs and management burdens for enterprises.

This study aims to deeply analyze the risks faced by the car rental industry from an enterprise perspective and propose an innovative early warning model for car rental contract breaches defaults. This model combines DBSCAN clustering, improved by the Snow Ablation Optimizer (SAO) algorithm, with iTransformer time series prediction, tailored to the temporal and spatial characteristics of various driving data. The DBSCAN clustering algorithm in the model does not require pre-specification of the number of clusters, can handle clusters of arbitrary shapes, and is insensitive to noise points, making it suitable for diverse vehicle data sets. The SAO algorithm dynamically adjusts DBSCAN’s clustering parameters, enhancing clustering accuracy and stability. The iTransformer model demonstrates superior performance in handling high-frequency and outlier data, particularly in predicting vehicle usage during peak hours and assessing default risk for long-distance rentals. Compared to traditional models such as ARIMA and LSTM, the iTransformer significantly enhances prediction accuracy and computational efficiency. By accurately forecasting the future trajectories of vehicles, this model facilitates real-time monitoring of customer defaults.

Despite extensive research on dynamic early warning and GPS trajectory prediction in fields such as shipping, taxis, and smart vehicles, existing studies on dynamic default early warning for vehicle leasing primarily rely on a combination of manual and background systems. This approach has notable limitations, including delayed response times and an inability to effectively prevent irreversible losses to enterprises and negative impacts on industry development. Furthermore, existing research on constructing electronic fences for leased vehicles mainly relies on trajectory data playback from background management systems, failing to fully leverage data model advantages and resulting in significant waste of human and system resources.

The SAO-DBSCAN model proposed in this paper determines the vehicle’s permanent residence, or electronic fence, through clustering analysis of GPS data and monitors dynamic changes in vehicle behavior in real time. The iTransformer model forecasts vehicle trajectories based on GPS data. When a vehicle enters a predefined hazardous zone, the system automatically triggers an alarm and notifies relevant personnel, thereby enhancing operational efficiency and standardizing the car rental industry.

The primary contributions of this paper are as follows:

1. The application of an algorithmic model to facilitate manual and background management system assessments significantly enhances operational efficiency and reduces the risk of triggering defaults, thereby improving customer satisfaction in the car rental industry.
2. The DBSCAN clustering model analyzes vehicle trajectory data, with the SAO algorithm dynamically adjusting clustering parameters to accurately determine the vehicle’s permanent residence. The electronic fence is delineated by the latitude and longitude of this residence.
3. The iTransformer model algorithm accurately and swiftly predicts vehicle trajectory data within the electronic fence, using objective sequence data to forecast future vehicle locations and prevent vehicles from entering dangerous areas such as borders and coastlines.
4. The dynamic algorithm-based early warning system for vehicle monitoring, based on the permanent location of the electronic fence, digitizes the monitoring of rented vehicles, reducing economic losses for car rental companies and significantly improving customer service efficiency and satisfaction.

## 2. Literature review

The growth in per capita car ownership and the demand for holiday travel have contributed to the rapid expansion of the car rental industry. However, this sector also faces a number of challenges, including customer defaults, fluctuations in service quality, and insufficient market supervision. These factors have constrained the industry’s ability to flourish in a sustainable manner. In order to address these challenges, Solomon [3] put forth a proposal for the implementation of a web-based and service-oriented rental system, with the aim of enhancing the efficiency and quality of rental services. Ren [4] proposed the establishment of a robust membership system and membership credit files, with the objective of cultivating a stable customer base with favorable credit ratings, thereby mitigating the risk of customer default. Postorino [5] employed blockchain technology to decentralize the car-sharing system, with the objective of facilitating customer-to-customer car rental and addressing the inherent risks associated with car rental at the source. Nevertheless, there is a dearth of empirical rental data, and the deployment of blockchain technology remains largely theoretical. This paper will utilize a default warning system that integrates clustering algorithms and neural networks to address the issue of customer default warnings.

In order to reduce the demand for human and computing resources, this study explores the potential application of vehicle electronic fence construction algorithms. An [6] put forth a parallel DBSCAN algorithm based on spatial-temporal random partitioning (STRP-DBSCAN) and a PER-SAC algorithm that fuses a priority experience replay mechanism with the SAC algorithm, which markedly enhances the clustering efficacy and precision of spatial-temporal trajectory data. Nevertheless, further enhancements are necessary to enhance the automatic adjustment and universality of unlabeled data, as well as the automatic adjustment and versatility. MENG [7] put forth an enhanced and adaptive DBSCAN algorithm to enhance the adaptability of the DBSCAN algorithm; however, the algorithm’s efficiency remains suboptimal. Bao [8] analyzed trajectory data from shared taxis using a DBSCAN clustering algorithm based on spatial-temporal density and provided recommendations for optimizing the layout of taxi stations. It has been demonstrated that the DBSCAN clustering algorithm is effective in processing spatial-temporal trajectory data. The SAO-DBSCAN algorithm, as proposed in this paper, is capable of calculating the permanent residence of vehicles through the analysis of historical trajectory data, subsequently dividing the electronic fence in accordance with the results. This approach not only reduces the cost of monitoring but also enhances the efficiency and accuracy of the early warning system. Furthermore, this paper considers the specific characteristics of vehicle driving data and employs a dynamic parameter adjustment process based on the SAO algorithm to align the electronic fence construction with the diverse driving trajectories of different vehicles.

In the field of rental default warning, existing research is primarily focused on the financial domain, employing loss models and machine learning algorithms to alert customers of potential defaults, thereby providing a research methodology for rental default warning. In a related study, Kabosy F [9] and colleagues investigated the loss given default (LGD) in the rental sector. They employed a range of parametric and non-parametric techniques to predict LGD and compared the predictive accuracy of these methods to identify the most effective prediction model. However, these models have inherent limitations in terms of their ability to monitor and prevent customer default behavior in real time. These limitations include slow response times, limited data processing capabilities, and poor adaptability to changing market environments. Stein et al. [10] discussed the complexity of default prediction model validation, emphasizing the importance of model validation, and proposed possible challenges and solutions in practical applications, including model overfitting, data quality issues, and the frequency of model updates.

In the context of machine learning, Bazan, F. [11] and others conducted a comparative analysis of the performance of various machine learning classifiers in the default prediction of small Italian companies. Their findings indicated that machine learning exhibited superior performance compared to traditional logistic regression in default prediction, achieving notable prediction outcomes. Korangi [12] and others investigated the default probability structure of medium-sized companies and employed a deep learning method based on a transformer model for prediction. Their findings demonstrated that the deep learning architecture markedly enhanced the prediction performance in comparison to traditional models. In a study utilizing a European dataset comprising millions of residential mortgages, Barbaglia [13] compared the efficacy of diverse machine learning algorithms and logistic regression in loan default prediction. The findings indicated that machine learning algorithms offer enhanced precision in this domain. Tsai [14] and others have enhanced the precision of loan default prediction and optimized risk management by employing diverse machine learning techniques, such as neural networks, to inform financial regulatory decisions.

While existing models focus on financial aspects and use machine learning for prediction, they often lack real-time monitoring capabilities and adaptability to diverse data. The aforementioned studies demonstrate that neural network algorithms are more effective in addressing default warnings. In consideration of the time series characteristics inherent to vehicle GPS data, this paper proposes the design of a time series prediction model for the purpose of trajectory prediction and the prevention of vehicle defaults. The majority of current research is based on models including RNN, GRU, and LSTM, but these models have been observed to perform differently when dealing with different types of time series data. At present, the LSTM model is the most frequently employed for trajectory prediction of a single vehicle. Deo et al. [15] put forth a vehicle trajectory prediction method based on LSTM, which employs the encoder to discern the dynamics of vehicle motion and utilizes the convolutional neural network to discern the interdependence of surrounding objects. Lin [16] put forth the STA-LSTM model, an extension of the LSTM model, which incorporates a spatiotemporal attention mechanism to enhance the model’s interpretability. Given the proclivity of LSTM models to overfitting and gradient disappearance, some scholars have put forth the use of Transformer models for the prediction of vehicle trajectories. In a related vein, Yu [17] put forth a spatiotemporal graph Transformer model that yielded promising outcomes in the domain of pedestrian trajectory prediction. Messaoud [18] integrated the attention mechanism with the LSTM model and conducted experiments to demonstrate the influence of diverse attention modules on the model’s performance. Furthermore, Transformer models have been widely applied in early warning systems. For example, the STTEWS system predicts thermal runaway risks in lithium-ion batteries, and the BERTtery model forecasts battery failures in electric vehicles, highlighting the effectiveness of Transformer models in system fault prediction. However, Transformer models do not account for the correlation between data points over time. This is particularly critical in the analysis of driving datasets, where the correlation between adjacent timestamps is often a key factor [19,20].

Accordingly, this paper puts forth the iTransformer model as a means of addressing the issue of correlation learning. The iTransformer model, developed by other research groups, introduces several enhancements over the conventional Transformer model. These improvements include an efficient attention mechanism that reduces complexity from quadratic to linear, making it more suitable for high-dimensional, multivariate time series data [21,22]. Additionally, by inverting the conventional Transformer structure, iTransformer better captures correlations between different variables, which is essential for time series forecasting. The model also employs a flexible training strategy that allows it to be trained on an arbitrary number of series, thereby improving its adaptability and efficiency [23]. Moreover, it demonstrates enhanced utilization of lookback windows, which improves its performance on long-term forecasting tasks [24]. The experimental results demonstrate that the iTransformer model exhibits superior accuracy compared to other time series models, and it also outperforms the conventional Transformer model in terms of effectiveness. The iTransformer model’s efficacy in addressing intricate time-dependent challenges makes it an optimal choice for capturing long-term trends and periodic patterns in the driving activities of individual vehicles. This renders it an ideal methodology for dynamic default early warning systems, particularly in scenarios where customer default risks necessitate real-time monitoring and warning.

In conclusion, the DBSCAN algorithm and the neural network algorithm demonstrate robust clustering and prediction capabilities when processing large-scale data sets. However, their application in vehicle dynamic default early warning remains limited. Accordingly, this paper integrates the DBSCAN algorithm with the iTransformer model to accommodate the trajectory data of disparate vehicles, thereby establishing an integrated prediction system. The DBSCAN-iTransformer (DI) model, as proposed in this paper, has been demonstrated to be an effective method for preventing customer defaults, thereby offering a robust risk management tool for car rental companies.

## 3. Methods

**Fig 1:**
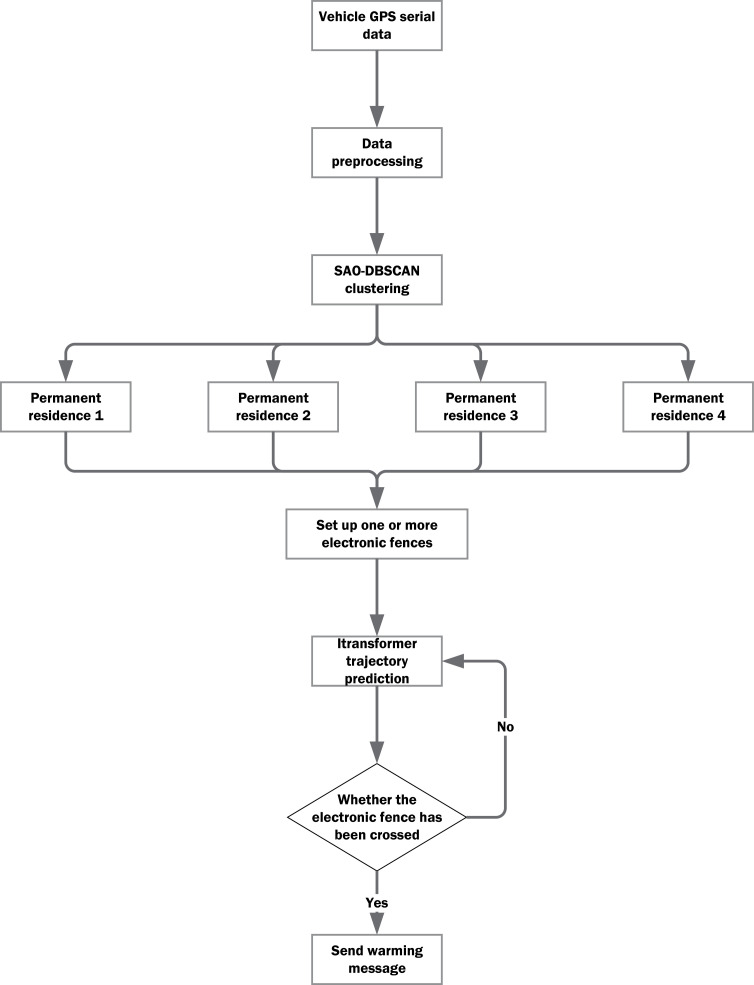
SAO-DBSCAN-iTransformer model flow chart.

This paper focuses on the dynamic prediction of vehicle defaults, introducing an innovative model based on the DBSCAN-iTransformer framework, as informed by existing literature. The model first determines the vehicle’s permanent residence using GPS data processed through the DBSCAN algorithm, subsequently constructing an electronic fence around this location. The iTransformer model is then employed to calculate the vehicle’s GPS data in real time, predicting the vehicle’s location for several time steps based on the reporting time of the vehicle’s device. To evaluate the efficacy of the proposed model, several alternative models were developed for comparison, including simple neural networks, GRU, LSTM, and Transformer models. Four actual vehicles were selected for testing to assess the model’s performance. A comprehensive set of evaluation metrics was employed, including vehicle accuracy, vehicle error (Error Range, unit: km), MAE, MSE, and RMSE. The efficacy and efficiency of the model will be further validated in a car rental company. The complete prediction model process is illustrated in Fig 1.

### 3.1. SAO-DBSCAN Model

The DBSCAN (Density-Based Spatial Clustering of Applications with Noise) algorithm is widely used for cluster analysis of vehicle GPS data. Spatial Clustering of Applications with Noise) algorithm is widely used because of its ability to detect clusters of arbitrary shape and to discriminate noisy points. However, the performance of the DBSCAN algorithm is highly dependent on two key parameters: the radius value and the minimum number of points. In order to improve the clustering effect of the DBSCAN algorithm, this paper introduces the Snow Ablation Optimizer (SAO) to optimize these parameters. The convergence speed in the DBSCAN clustering process is dynamically adjusted by the SAO algorithm to achieve the purpose of fast and accurate clustering. The following is the process of the specific two algorithms.

The Snow Ablation Optimizer (SAO) is a novel meta-heuristic algorithm that simulates the sublimation and melting of snow in nature. The SAO algorithm primarily simulates the sublimation and melting behavior of snow in order to achieve a balance between exploitation and exploration in the solution space, thereby preventing premature convergence of the algorithm [25,26]. In the field of complex problem solving, multi-objective optimization problems have attracted considerable attention due to the constraints they present. In contrast to single-objective optimization problems, which typically converge on a single optimal solution, multi-objective optimization allows for the identification of a range of potential solutions. The SAO algorithm has been designed to address this type of problem [5]. The fundamental principle underlying the SAO algorithm is the simulation of sublimation and melting processes observed in snow. In the natural world, the melting of snow is a process that involves two distinct phases: sublimation (the direct transformation of a solid into a gas) and melting (the transformation of a solid into a liquid). This process is employed in the algorithm to achieve a balance between exploration and exploitation, thereby enabling the identification of the optimal solution in a global optimization problem. The model flow of the SAO algorithm can be expressed as follows:

The initialization of the snow particle swarm is a fundamental step in the process. The initialization of a snow particle swarm is a prerequisite. Each particle represents a potential solution in the solution space. The position of the exemplar at time t is represented by the vector ${\text{x}}_{\text{i}}(t)$.

The fitness function is defined as follows: The fitness of each particle is calculated by the function $\text{f}(x_{i}(t))$, which evaluates the quality of the particle’s position.

Position Update: The position of the particle is updated by the sublimation and melting processes, which can be expressed as follows:


$${\text{x}}_{i}(t+1)=x_{i}(t)+\Delta x_{sub}(t)+\Delta x_{melt}(t),$$ (1)

Where, $\Delta x_{sub}(t)$ is the position change caused by sublimation, while $\Delta x_{melt}(t)$ is the position change caused by melting.

The sublimation process is defined as the transformation of a solid substance into a gas without passing through a liquid state. The sublimation process is a simulation of the phenomenon whereby snow directly transforms from a solid state to a gaseous state. This process can enhance the exploration capacity of particles. The formula is as follows:


$$\Delta x_{sub}(t)={\text{r}}_{1}\cdot ({\text{x}}_{gbest}-x_{\text{i}}(t))$$ (2)

Where ${\text{x}}_{gbest}$ is the current global optimal solution position, and ${\text{r}}_{1}$ is a random number used to simulate randomness in nature.

The melting process is defined as the transformation of a solid into a liquid state. The melting process is a phenomenon that simulates the transformation of a solid into a liquid. This process can enhance the development capacity of particles, and the formula is as follows:


$$\Delta x_{melt}(t)={\text{r}}_{2}(x_{pbest,i}-x_{i}(t))$$ (3)

Where $x_{pbest,i}$ is the particle i’s historical optimal position, which ${\text{r}}_{2}$ is also a random number.

Algorithm Termination Conditions: The algorithm continues to iterate until the termination conditions are met, such as reaching the maximum number of iterations or the quality of the solution no longer improving significantly.

The DBSCAN algorithm is a type of density clustering algorithm. The GPS data of vehicles exhibits both time series and density spatial characteristics. Consequently, the DBSCAN algorithm is a more effective means of extracting the spatial characteristics of the GPS data set, thereby enabling the identification of the vehicle’s permanent residence. The objective of the DBSCAN algorithm is to identify clusters of any shape in any dataset, while distinguishing between noise points, i.e., data anomalies. The DBSCAN algorithm incorporates the radius value and the minimum number of points within the Eps radius, MinPts. The algorithm is defined as follows:

Domain: *δ* - neighborhood of data point x, denoted as $N_{\delta }\text{(x)}$, is the set of all points within a specified radius around point X. The mathematical expression is as follows:


$$N_{\delta }\text{(x)}=\{y\in D|d(x,y)\leq \delta \}$$ (4)

where d is a distance measure belonging to the set of positive real numbers R + . According to this definition, the point X is always part of its own domain, $\text{x}\in N_{\delta }(x)$ always true. The size of the domain is $\left|N_{\delta }\text{(x)}\right|$.

Classification of points: in dataset D can be classified as: core points, boundary points, and noise points. If the -neighborhood density of point x is high, that is $\left|N_{\delta }\text{(x)}\right|$> MinPts, where MinPts is the density threshold specified by the user, then the point is considered a core point. If point x is not a core point, but the point is within the *δ* -neighborhood centered on the core point, then the point is considered a boundary point. The rest are boundary points.

Direct density up to: If the point q in dataset D can be reached directly from point p, the following conditions must be met:


$$\left|N_{\delta }\text{(p)}\right|\;\geq \text{ minPts}$$ (5)

Density up to: If the points p in the dataset D are reachable from the point q, there is a sequence (x_1_, x_2_,..., x_n_) in D such that q =  x1 and p =  xn. Two points in the sequence are considered to be reachable if they are consecutive.

Density-based clustering: A density-based cluster C is a non-empty subset of the dataset D that satisfies the following condition: if any point p and q in the cluster are reachable from p, then q also belongs to cluster C. Any point p and q in the cluster are connected to each other by density.

**Fig 2:**
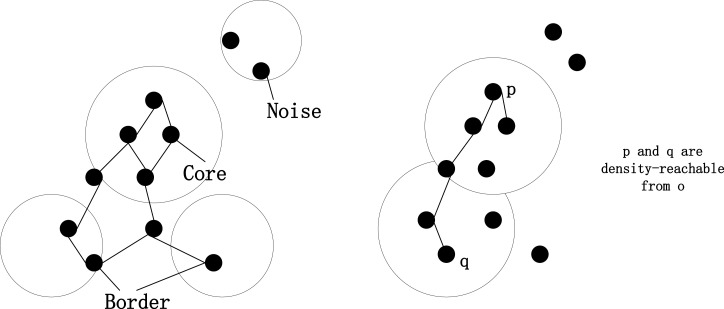
Flow chart of the DBSCAN algorithm.

The core idea of the DBSCAN algorithm is to gradually build clusters by exploring the core points and their *δ* -neighborhoods in the data space. Specifically, the algorithm randomly selects a point p from the dataset and checks the number of points in its *δ* -neighborhood. If the number reaches the preset minimum point (MinPts) threshold, point p is considered a core point and a new cluster is formed based on it. The algorithm then includes all points in the *δ* -neighborhood of p in the cluster and expands the cluster by exploring the -neighborhoods of these points in the same way. If no new core points are found during the expansion process, the cluster is complete. The algorithm continues to process the remaining points until all core points have been explored, resulting in multiple independent clusters. Points that are not assigned to any cluster are considered noise. The algorithm process is shown in Fig 2:

### 3.2. iTransformer Model

Multivariate time series forecasting aims to predict the target values of future time steps by extracting the time features of historical multivariate observations. The superior performance of the iTransformer in specific scenarios is primarily due to its advanced attention mechanism. This mechanism allows the iTransformer to effectively focus on relevant features while ignoring noise in high-dimensional data and complex temporal patterns. For instance, when processing datasets with significant noise and outliers, the iTransformer can identify and filter out these disturbances, thereby enhancing prediction accuracy and stability. In contrast, traditional models often struggle with similar data due to their lack of effective feature selection and noise filtering capabilities. The iTransformer model designed in this paper adopts the pure encoding architecture of the Transformer, including embedding, projection, and Transformer modules [27]. Unlike traditional Transformer, iTransformer embeds variables as tokens and applies self-attention mechanism to capture the complex dependencies between them. In addition, iTransformer also considers the time lag between variables and the differences between different physical measurements. This makes iTransformer excel in multi-variable prediction tasks. It is also faster than traditional Transformer models and more accurate.

The iTransformer model first embeds each variable of the time series independently into a token. Let there be a collection of time series, where each sequence consists of multiple variables, denoted as X =  {x1, x2,..., xT}, where T represents the length of the vehicle GPS sequence in this paper, and Xt is the vector of variables at time point t. The model algorithm process is as follows:

Embedding: The entire time series of each variable is embedded into a token. E =  Embedding(X), where E is the representation after embedding.

Self-Attention: The iTransformer model uses an attention mechanism to capture the correlation between variables. The calculation of the self-attention mechanism can be expressed as follows:


$$Attention\left(Q,K,V\right)=\text{so}ft\max(\frac{QK^{T}}{\sqrt{d_{k}}})v$$ (6)

Where Q, K, and V are the query, key, and value matrices, respectively, and dk is the dimension of the key vector.

Feed-Forward Network: For each variable token, the iTransformer model uses a feed-forward neural network to learn a nonlinear representation, which can be expressed as:


$$FNN(\text{x)}=ReLU(xw_{1}+b_{1})w_{2}+b_{2}$$ (7)

where w1, w2 are the weight matrices, b1, b2 are the bias terms, and ReLU is the activation function.

Layer normalization: In iTransformer, layer normalization is used for the time series representation of a single variable to reduce differences caused by inconsistent measurements.

Projection: Finally, the model outputs a prediction by projecting.


y_pred=Projection(E) (8)

Where y_pred is the predicted time series.

The iTransformer model’s structure enables it to effectively process time series data and achieve excellent performance in multivariate prediction tasks [28]. The iTransformer model not only learns the temporal characteristics of vehicle GPS data but also handles the linear and non-linear relationships between multiple features of the vehicle GPS data set. It is therefore able to accurately predict the location of the vehicle at future time steps. The model provides effective evidence for managers to prevent vehicles from exceeding the electronic fence.

## 4. Experimental design

The experiments in this paper are designed around the GPS latitude and longitude data of vehicles, and a model comparison experiment is designed. The benchmark models used in the model comparison experiment include VAR, BP neural network, GRU network model, LSTM network, and transformer model. The experiments are used to verify the completeness and scientific of the model system. In order to improve the reliability and generalization ability of the model, the K-fold cross-validation method is introduced in the experiment. Specifically, the data set is divided into K subsets, and each time a model is trained using K-1 subsets and tested using the remaining subset, this is repeated K times, and the final average is taken as the performance indicator of the model. The evaluation indicators designed in this experiment include the residence index and the time series evaluation index. Among them, the residence index includes the error range (km) and calculation time, etc.; the time series evaluation index includes the MAE, MSE, and error range (km), etc. The experimental study found that the model designed in this paper has good performance and high accuracy in all indicators.

### 4.1. Data source

**Fig 3:**
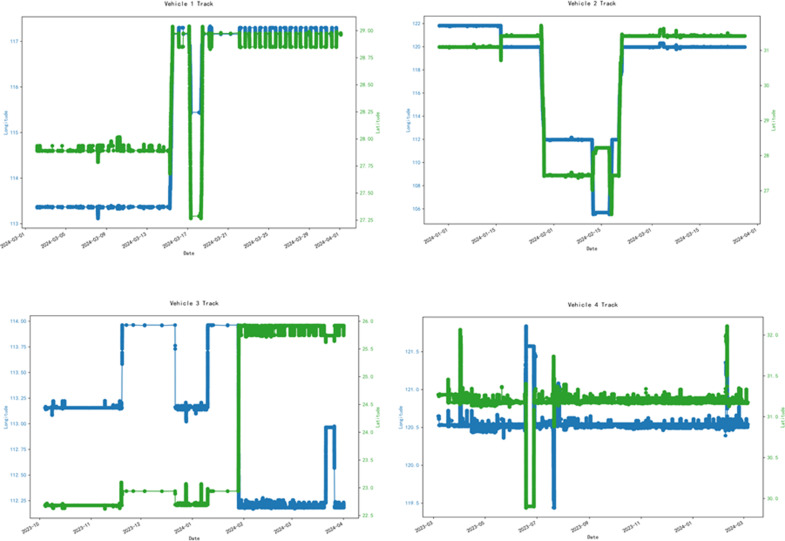
Vehicle driving data chart.

The data in this article comes from the GPS data of actual rented vehicles provided by NASDAQ-listed car rental companies. All data has been processed to remove sensitive customer information, and the data set only includes the GPS track data generated by the vehicle during driving. In this experiment, vehicles with different rental periods are used as experimental objects to verify the scientific validity of this model. As shown in Fig 3, the rental periods of the vehicles are one month, three months, six months, and one year. According to the different driving habits of each owner, the driving data also varies greatly. Therefore, the DBSCAN-iTransformer model is designed to learn the driving habits of each owner based on the characteristics of each driving data, thereby achieving the ability of accurate prediction. The specific data range of each vehicle is shown in Table 1:

**Table 1:** Time span of vehicles.

Vehicle type	Vehicle 1	Vehicle 2	Vehicle 3	Vehicle 4
Time span	2024/03/01-2024/04/01	2023/12/29-2024/03/28	2023/10/04-2024/04/01	2023/03/06-2024/03/06
Data length (rows)	4439	128418	163508	1048576

### 4.2. Data preprocessing

(1) Data Sampling

Given that the frequency of vehicle data reporting varies, this paper employs a sampling frequency of one minute for the data set, based on actual driving habits.

(2) Data Cleaning and Division

I In this experiment, the anomalous latitude and longitude data will be corrected directly, and the missing values will be replaced with the mean value. This paper divides the dataset into two categories: one constructs the residence through the DBSCAN algorithm, and the other predicts the latitude and longitude of the vehicle through the iTransformer model.

(3) Data normalization

It should be noted that different evaluation metrics often have different dimensions and dimensional units. To mitigate the impact of dimensionality, the data is normalized by mapping the original driving data set to a specific range. Furthermore, the detrimental effects of a single data sample can be mitigated [15]. The data normalization equation is as follows:


$$z_{n}=\frac{z_{i}-z_{\min}}{z_{\max}-z_{\min}}$$ (9)

where n is the sample size; is the normalized data; is the original data; and are the minimum and maximum values of the vehicle driving sequence data, respectively. The prediction results of each dataset based on the iTransformer model are summarized, and then the resulting dataset is de-normalized using Equation 10.


$$f_{i}=f_{n}(z_{\max}-z_{\min})+z_{\min}$$ (10)

(4) Data Partition

In this paper, the normalized driving latitude and longitude data set is divided according to one-fifth of the data length, with 80% of the data used as the training set and 20% as the test set. The training set is employed to train the model and identify optimal model parameters, while the test set is utilized to assess the model’s accuracy and efficiency. In this study, the sequence length was set to 60, the prediction sequence length was set to 10, and the historical data from the previous hour was used to predict the latitude and longitude data 10 minutes later.

### 4.3. Experimental evaluation indicators

The prediction error in the analysis test set is evaluated using the MAE, MSE, MAPE and Accuracy indicators. The lower the three indicators, the lower the model error. Indicator 14 intuitively represents the distance between the predicted value and the true value on the soil surface. The formula for calculating the distance is shown in 13, and the specific calculation formula is as follows:


$$MAE=\frac{1}{n}\sum_{t=1}^{n}\left|A_{t}-F_{t}\right|$$ (11)

$$MSE=\frac{1}{n}\sum_{t=1}^{n}\left(A_{\text{t}}-F_{t}\right)$$ (12)

$$\text{d=2r}\cdot \text{arcsin(}\sqrt{{\text{sin}}^{2}\text{(}\frac{\Delta \phi }{2}\text{)+cos(}\phi_{1}\text{)}\cdot \text{cos(}\phi_{2}\text{)}\cdot {\text{sin}}^{2}\text{(}\frac{\Delta \lambda }{2}\text{)}}\text{)}$$ (13)

Accuracy=geodesic(actual,pred) (14)

$$MAPE=\frac{100\%}{\text{n}}\sum_{t=1}^{n}\left|\frac{A_{t}-F_{t}}{A_{t}}\right|$$ (15)

Where At is the actual value at time t; Ft is the predicted value at time t; d is the distance between two points(r) is the radius of a sphere (for example, the radius of the Earth is about 6371 kilometers), $\phi_{1}$ and $\phi_{2}$ are the latitudes of two points, Δλ and Δϕ are the longitudes of two points actual represents the actual position of the vehicle; Actual and Pred represent the actual and predicted latitude and longitude values, respectively.

### 4.4. Experimental parameter settings

The benchmark models for the experiment’s resident judgment module include K-Means, KDE kernel density estimation, Spectral clustering, and Agglomerative clustering. The default parameters are used for the K-Means and KDE kernel density estimation algorithms. For Spectral clustering, the parameters are set with affinity using nearest neighbors, the number of neighbors set to 10, and the number of clusters determined dynamically based on the data. For Agglomerative clustering, the linkage method is Ward, the distance metric is Euclidean, and the number of clusters is also determined dynamically based on the data. The benchmark models for the experiment’s prediction module are VAR, BP, GRU, LSTM, and Transformer. The parameters of the VAR model are primarily determined automatically by the model’s fit() function, with no explicit parameter settings. During fitting, the VAR model automatically selects the optimal number of lags based on the data. The BP neural network model parameters are set to two hidden layers, a learning rate of 0.01, 50 iterations, an input length of 60, and an output of 10. The GRU model parameters are set to two hidden layers with 256 neurons per layer and 50 training iterations, identical to the LSTM model parameters. The optimizer used is Adam. The Transformer model parameters are set to an input size of 2, representing latitude and longitude, respectively, with an 8-layer, 4-head, feed-forward network, a 4-fold amplification factor, and a dropout rate of 0.1 to prevent overfitting.in the SAO-DBSCAN-ITransformer model designed in this study, the parameters of the SAO algorithm are set to an eps range of (0.01, 1), a minPts range of (2, 200), and 100 iterations. The parameters of the DBSCAN model are determined by the SAO algorithm. The specific parameters are shown in Table 2. The parameters of the iTransformer model are the same as those of the Transformer model described above.

**Table 2:** DBSCAN model parameter settings.

Vehicle model	Car 1	Car 2	Car 3	Car 4
(eps,minPts)	(0.047,103)	(0.05, 100)	(0.053, 98)	(0.05,100)

## 5. Results

In this study, we used the SAO-DBSCAN algorithm to construct electronic fences for vehicles, and compared K-Means KDE, Agglomerative, Spectral, The performance of the SAO-DBSCAN five algorithms in vehicle trajectory prediction. The experimental results are shown in Table 3.

**Table 3:** Comparison of algorithms for vehicle home location.

Vehicle 1	K-Means	Agglomerative	Spectral	KDE	SAO-DBSCAN
Running time (s)	9.81	12.34	15.67	458.27	**3.27**
Predicted location (GPS)	(119.553538, 31.176192)	(119.560000, 31.180000)	(119.570000, 31.185000)	(119.9722525, 31.406777)	**(119.977215, 31.409117)**
Accuracy(KM)	48.00	35.00	30.00	1.5	**0.08**

The results indicate that the SAO-DBSCAN algorithm consistently outperformed other clustering methods in terms of accuracy and running time. Specifically, the SAO-DBSCAN algorithm achieved the highest accuracy with an error of only 0.08 km, significantly lower than the errors observed with other algorithms. This superior performance can be attributed to the dynamic adjustment of clustering parameters by the Snow Ablation Optimizer, which enhances both clustering accuracy and stability. In contrast, traditional clustering algorithms such as K-Means and Agglomerative clustering exhibited significantly higher error rates, with K-Means showing an error of 48.00 km and Agglomerative clustering 35.00 km. These methods also demonstrated longer running times compared to SAO-DBSCAN, highlighting their inefficiency in handling vehicle GPS data. Spectral clustering showed moderate performance with an error of 30.00 km, but it also required more computational resources compared to SAO-DBSCAN. Kernel Density Estimation (KDE), while more accurate than K-Means and Agglomerative clustering, still fell short of the performance of SAO-DBSCAN and had a notably long running time of 458.27 seconds, which limits its practical application despite its relatively higher accuracy.

Overall, the SAO-DBSCAN algorithm demonstrated the best balance between accuracy and computational efficiency, making it the most suitable choice for real-time vehicle default prediction in the car rental industry. However, it is important to consider the stability of the SAO-DBSCAN algorithm under varying conditions to ensure consistent performance.

**Fig 4:**
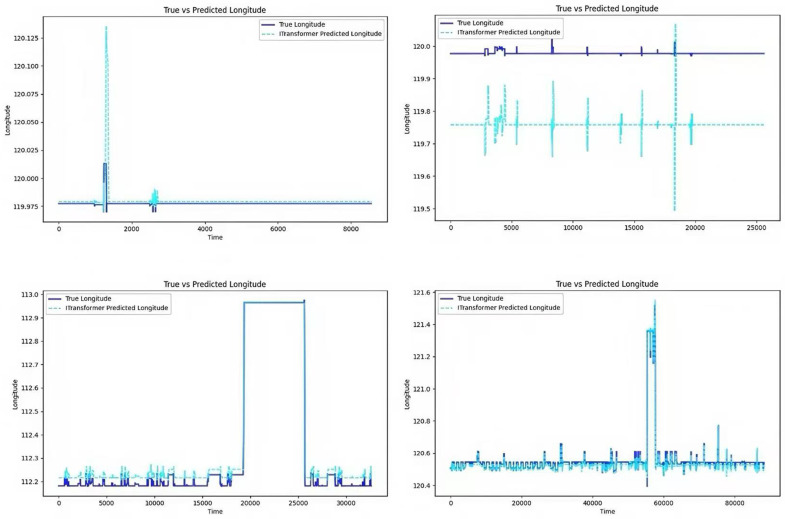
Comparison of model predictions for longitude.

Once the electronic fence of the vehicle has been obtained, the vehicle within the electronic fence is monitored, and the vehicle’s driving data, specifically its latitude and longitude data, is predicted for future time periods using the iTransformer model. The experimental model is exemplified by the vehicle within the vehicle fence 1, which has three characteristics: time, longitude, and latitude. The output is comprised of two features, namely longitude and latitude. Fig 4 illustrates the comparison between the predicted longitude of the model and the real longitude. The model demonstrates a high degree of accuracy in trend prediction, with minimal error in distance between the surface and the real longitude, and a curve that closely approximates the real value.

**Fig 5:**
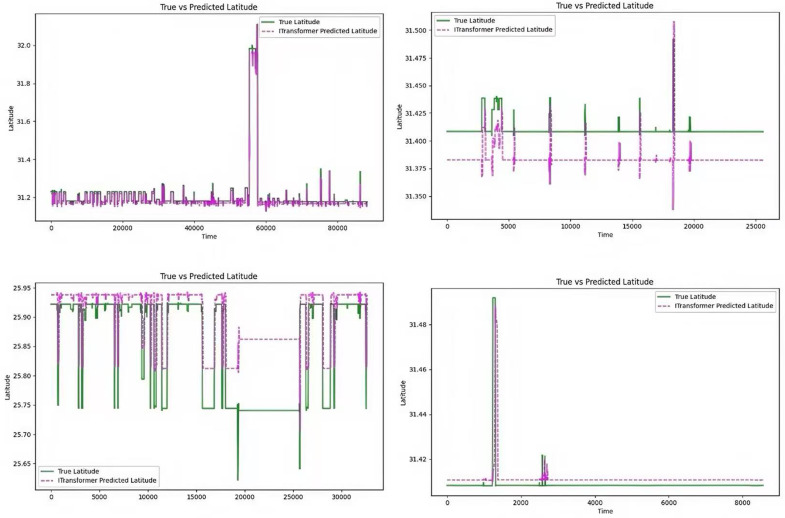
Comparison of model predictions for latitude.

The comparison between the predicted latitude and the actual latitude is shown in Fig 5: the trend of the predicted latitude is consistent with the actual latitude fluctuation, and the accuracy is relatively high. However, compared with the longitude prediction, the latitude prediction has a certain lag, which leads to an error in the prediction accuracy compared with the longitude prediction.

**Fig 6:**
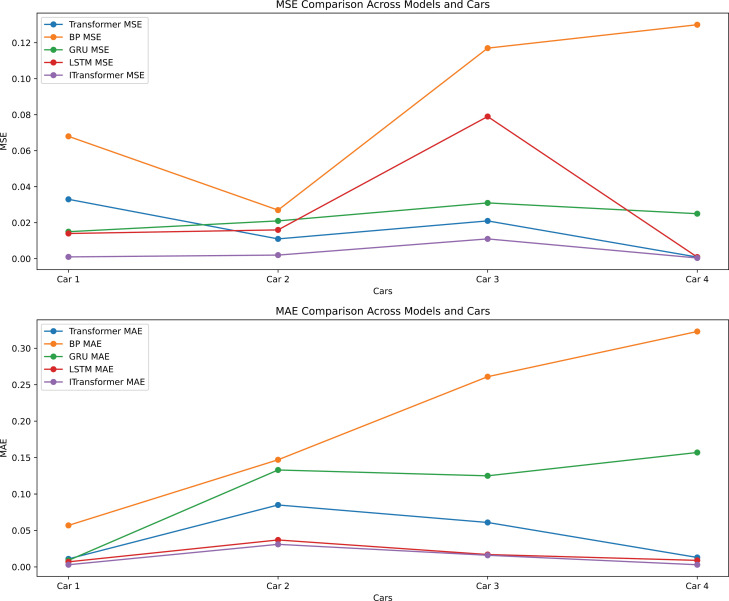
Model error comparison.

To ascertain the high degree of accuracy and scientific validity of this model, a comparative model was employed to corroborate the experimental outcomes. The experimental results, as illustrated in Fig 6, clearly demonstrate that the prediction errors (MSE and MAE) of the iTransformer model across different vehicles are significantly lower than those of other models. This indicates that the iTransformer model excels in capturing data characteristics and processing complex time series data. Specifically, the advanced attention mechanism of the iTransformer allows it to focus more effectively on relevant features while ignoring noise, thereby enhancing prediction accuracy and stability. Furthermore, the line chart in Fig 6 reveals the performance disparities among different models for each vehicle. Although the Transformer and LSTM models perform better on certain vehicles, they do not surpass the overall performance of the iTransformer model. The BP and GRU models exhibit larger errors, highlighting their limitations in handling high-dimensional data and complex temporal patterns.

In summary, the superior performance of the iTransformer model in vehicle trajectory prediction has been thoroughly validated, underscoring its practical application value in reducing prediction errors and improving accuracy. These findings not only emphasize the potential of the iTransformer model in complex time series data processing but also provide a robust theoretical foundation and practical support for future applications in vehicle trajectory prediction.

The specific comparison results are presented in Table 4. The iTransformer model demonstrated superior performance compared to other models on all vehicle datasets. A 5-fold cross-validation method was employed in the experiment to ensure the reliability and generalizability of the results.

**Table 4:** Comparison of trajectory prediction model results.

cars	error indicator	Transformer	BP	GRU	LSTM	iTransformer	CV Mean	CV Std Error
Car 1	MAE	0.011	0.057	0.009	0.007	**0.003**	**0.009**	**0.002**
	MSE	0.033	0.068	0.015	0.014	**0.001**	**0.026**	**0.005**
	MAPE(%)	0.057	0.143	0.121	0.032	**0.034**	**0.077**	**0.015**
Car 2	MAE	0.085	0.147	0.133	0.037	**0.031**	**0.087**	**0.020**
	MSE	0.011	0.027	0.021	0.016	**0.002**	**0.015**	**0.004**
	MAPE(%)	0.107	0.283	0.034	0.083	**0.050**	**0.111**	**0.025**
Car 3	MAE	0.061	0.261	0.125	0.017	**0.016**	**0.096**	**0.022**
	MSE	0.021	0.117	0.031	0.079	**0.011**	**0.052**	**0.012**
	MAPE(%)	0.147	0.324	0.302	0.180	**0.108**	**0.212**	**0.045**
Car 4	MAE	0.013	0.323	0.157	0.009	**0.003**	**0.101**	**0.023**
	MSE	0.0009	0.130	0.025	0.0009	**0.0004**	**0.031**	**0.007**
	MAPE(%)	0.030	0.829	0.302	0.018	**0.021**	**0.240**	**0.050**

With regard to the three indicators MAE, MSE, and MAPE, the error values of the iTransformer model are markedly lower than those of other models, particularly in the Car 1 and Car 4 datasets, where the iTransformer model exhibits superior performance. In the Car 1 dataset, the mean absolute error (MAE) value of the iTransformer model is 0.003, which is significantly lower than that of the Transformer (0.011), bidirectional long short-term memory (BLSTM) (0.057), gated recurrent unit (GRU) (0.009), and long short-term memory (LSTM) (0.007) models. Similarly, the MSE value of the iTransformer model is 0.001, which is also significantly lower than that of the other models. Moreover, the iTransformer model demonstrated remarkable proficiency in the MAPE metric, with a score of merely 0.034%. In cross-validation, the iTransformer model exhibited an average error (CV Mean) of 0.009 and a standard error (CV Std Error) of 0.002.

In the Car 2 dataset, the mean absolute error (MAE) value of the iTransformer model is 0.031, which is significantly lower than the values obtained by the Transformer (0.085), the bidirectional pipeline (0.147), the gated recurrent unit (GRU) (0.133), and the long short-term memory (LSTM) (0.037) models. Similarly, the mean square error (MSE) value of the iTransformer model is 0.002, which is also significantly lower than that of the other models. With regard to MAPE, the iTransformer model demonstrated superior performance, with a MAPE of only 0.050%. In cross-validation, the iTransformer model exhibited an average error (CV Mean) of 0.087 and a standard error (CV Std Error) of 0.021.

In the Car 3 dataset, the mean absolute error (MAE) value of the iTransformer model is 0.016, which is significantly lower than the values obtained by the Transformer (0.061), bidirectional propagation (0.261), gated recurrent unit (GRU) (0.125), and long short-term memory (LSTM) (0.017) models. Similarly, the MSE value of the iTransformer model is 0.011, which is also significantly lower than that of the other models. With regard to MAPE, the iTransformer model demonstrated superior performance, exhibiting a MAPE of only 0.108%. In cross-validation, the iTransformer model exhibited an average error (CV Mean) of 0.096 and a standard error (CV Std Error) of 0.023.

In the Car 4 dataset, the mean absolute error (MAE) of the iTransformer model is 0.003, which is significantly lower than the 0.013 of the Transformer, 0.323 of the BP, 0.157 of the GRU, and 0.009 of the LSTM. Similarly, the mean square error (MSE) of the iTransformer model is 0.0004, which is also significantly lower than that of the other models. Moreover, the iTransformer model demonstrated remarkable proficiency in the MAPE metric, attaining a score of merely 0.021%. In cross-validation, the iTransformer model exhibited an average error (CV Mean) of 0.101 and a standard error (CV Std Error) of 0.024.

The results demonstrate that the iTransformer model exhibits a notable advantage when processing vehicle datasets, with the capacity to more accurately predict vehicle performance indicators. This superiority is primarily attributable to the iTransformer model’s robust capacity to discern data characteristics and process intricate time series data. The incorporation of a self-attention mechanism allows the iTransformer model to more efficiently capture long-range dependencies in the data, thereby improving the precision of the prediction. Moreover, the iTransformer model utilizes a multi-level feature extraction approach during training, which facilitates more comprehensive data characterization, thereby enhancing the model’s performance.

## 6. Conclusions

This study proposes a vehicle rental early warning model based on an improved DBSCAN electronic fence and iTransformer trajectory prediction. The objective is to mitigate the economic losses incurred by customer defaults, vehicle idleness, and service quality degradation in the car rental industry. The efficacy and precision of the model in forecasting the vehicle’s future location and home location were validated through an analysis of the car rental market data provided by a company in China.

The experimental results demonstrate that the iTransformer model exhibits superior performance in predicting vehicle trajectories, with significantly lower MSE, MAE, and position error than the traditional model. This result demonstrates the iTransformer model’s capacity to process complex time series data and capture data characteristics. Furthermore, the DBSCAN clustering algorithm is capable of accurately identifying the home locations of vehicles and dividing the electronic fence through the clustering analysis of vehicle GPS data, thereby enabling real-time monitoring of vehicle behavior.

### The principal contributions of this study are as follows

An innovative vehicle rental early warning model was proposed, which combined the improved DBSCAN clustering algorithm based on SAO and the iTransformer trajectory prediction model to significantly enhance data processing capabilities and risk warning efficiency. By accurately identifying the permanent residence of the vehicle and predicting its future location, customer default behavior can be effectively prevented, thereby reducing the economic losses of the rental company. The implementation of digital monitoring of rented vehicles has resulted in a reduction in the cost of manual supervision, an improvement in service quality, and an enhancement in customer satisfaction. The model provides substantial support for the sustainable development of the car rental industry and has significant practical application value.

Although this study has yielded significant results, there are still some limitations to be considered. Firstly, the performance of the model may vary depending on the specific dataset used. Future research may seek to further optimize the model structure and parameters in order to enhance its applicability in a wider range of application scenarios. Secondly, this study is primarily based on the car rental market data of a company in China. In the future, it would be beneficial to consider incorporating additional data sources in order to verify the universality and robustness of the model. Moreover, vehicle rental markets in different geographic regions exhibit distinct characteristics and behavior patterns, which may influence the model’s prediction performance. Consequently, future research should focus on validating the model across various geographic regions and diverse datasets to ensure its wide applicability and robustness. Finally, the trajectory prediction in this study is limited to the driving data of the current vehicle. To achieve more accurate vehicle trajectory prediction, it is necessary to incorporate the driving data of other vehicles into the analysis, thereby reducing the impact of other vehicles on the research vehicle. This approach has been demonstrated to be effective in previous studies, including those referenced in [29,30,31].

## Supporting information

S1 Data (GZ)

S2 Data (GZ)

S3 Data (GZ)

S4 Data (GZ)

S5 Data (ZIP)
