# Supplementary figures and images for: An enhanced iTransformer-based early warning system for predicting automotive rental contract breaches
Source: PLoS One. 2025 Mar 20;20(3):e0319786. doi: 10.1371/journal.pone.0319786 (PMC11925311; doi:10.1371/journal.pone.0319786)

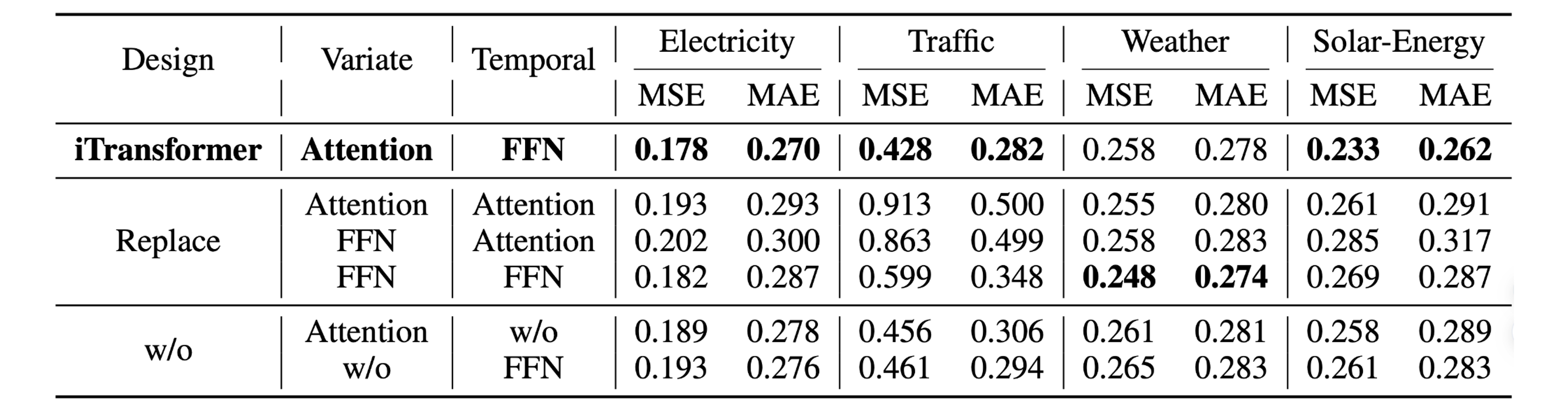

Supplement: S5 Data — (ZIP) [file pone.0319786.s005.zip › iTransformer-main/figures/ablations.png]

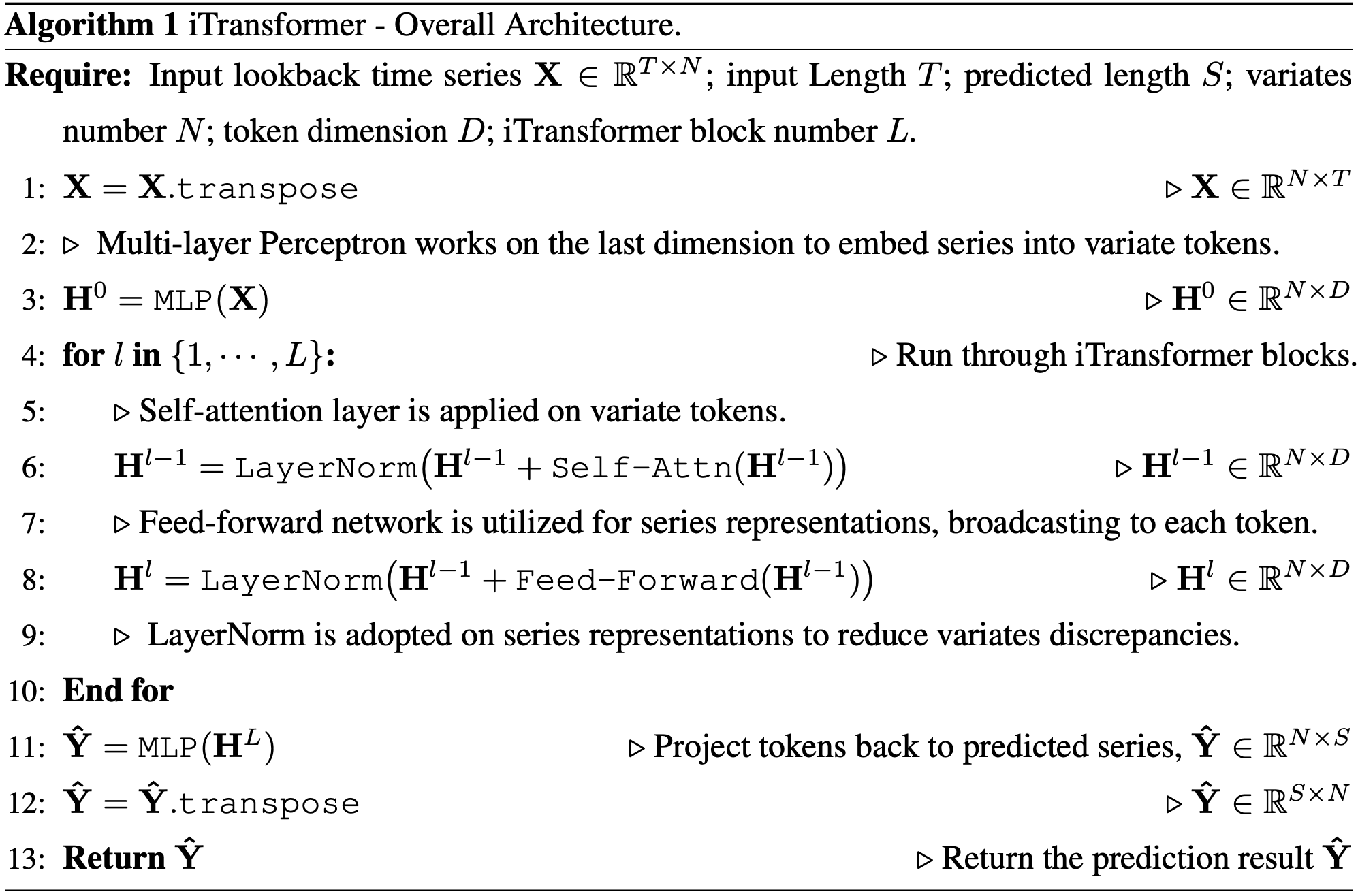

Supplement: S5 Data — (ZIP) [file pone.0319786.s005.zip › iTransformer-main/figures/algorithm.png]

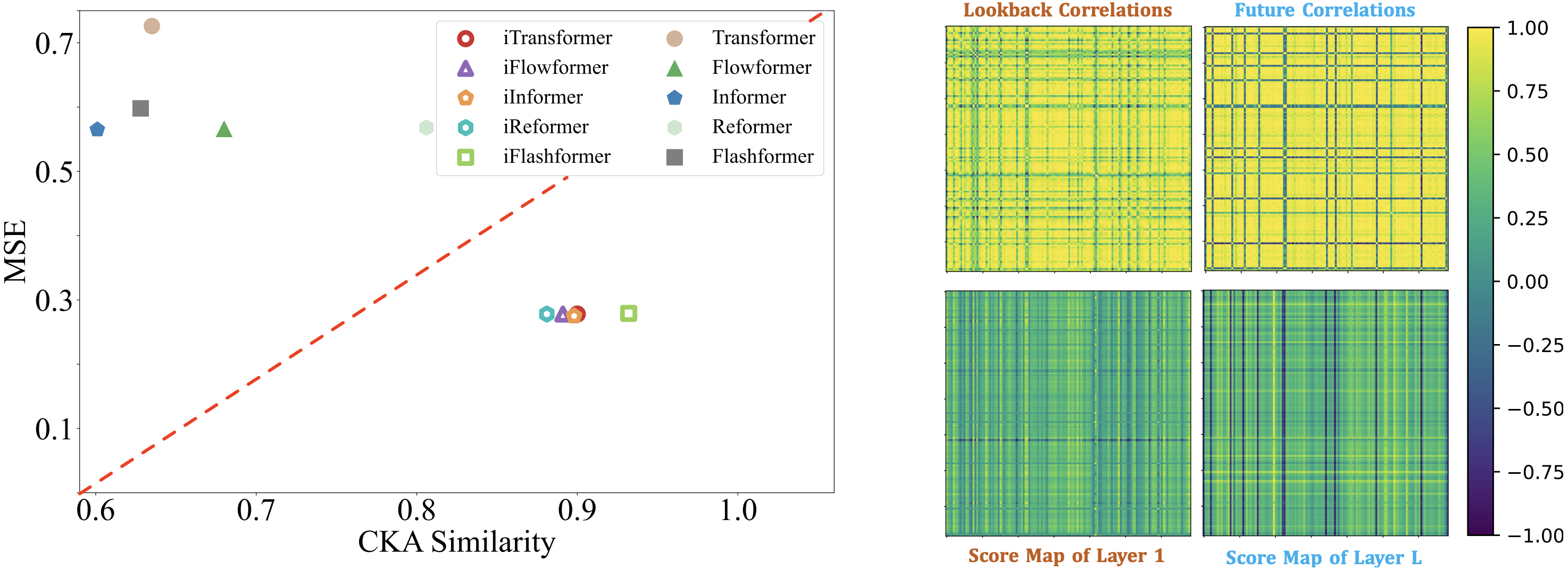

Supplement: S5 Data — (ZIP) [file pone.0319786.s005.zip › iTransformer-main/figures/analysis.png]

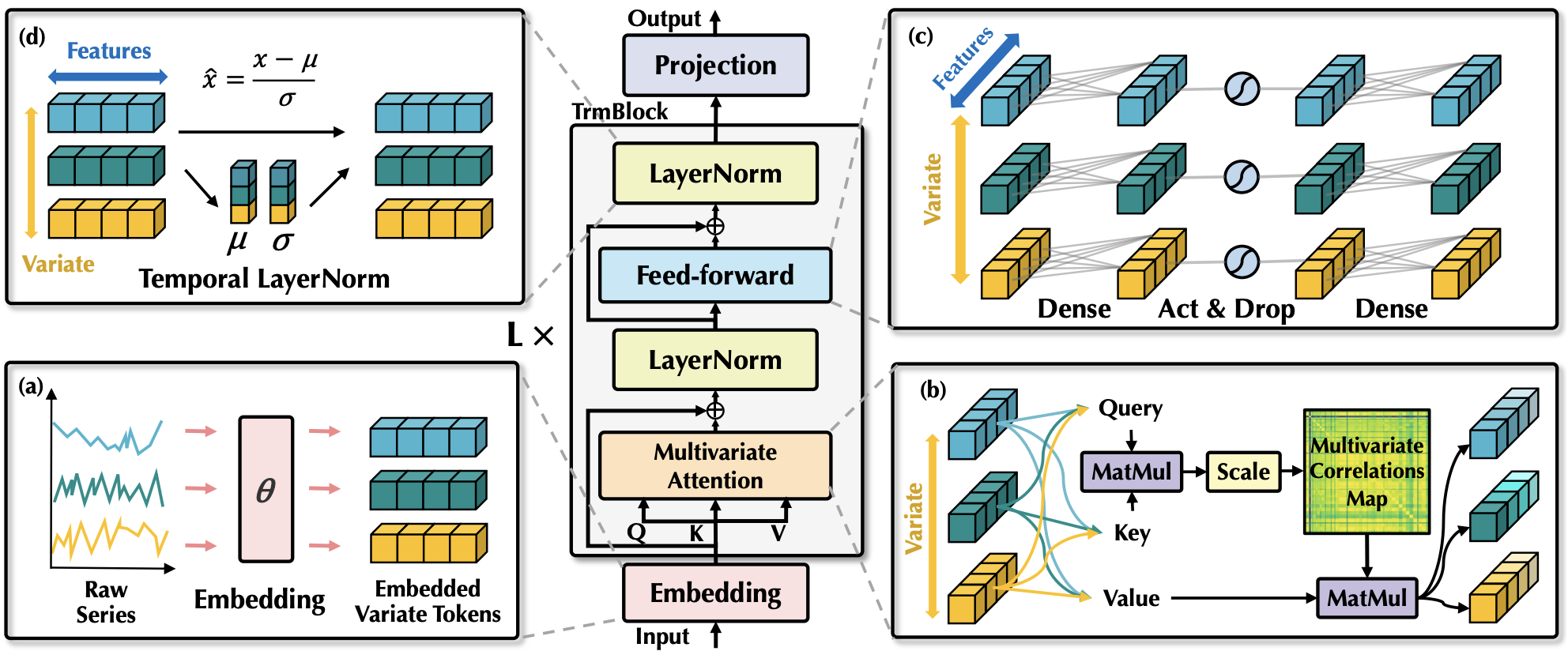

Supplement: S5 Data — (ZIP) [file pone.0319786.s005.zip › iTransformer-main/figures/architecture.png]

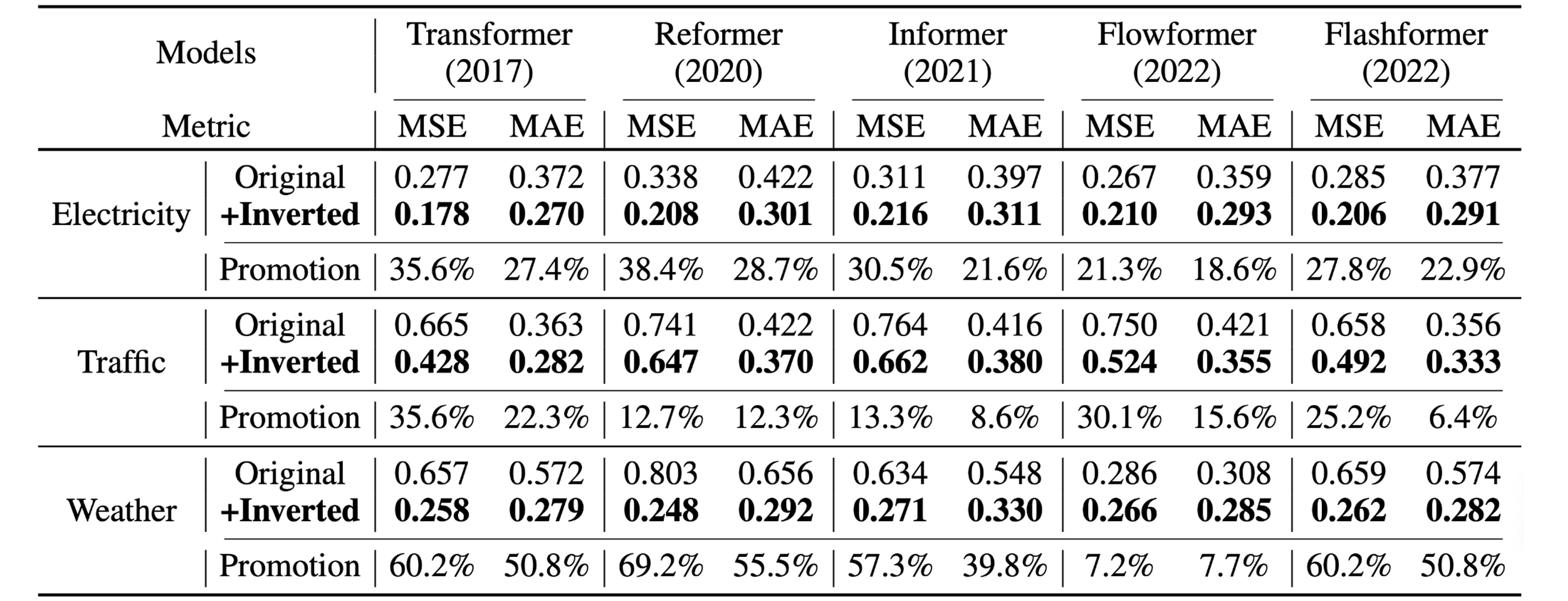

Supplement: S5 Data — (ZIP) [file pone.0319786.s005.zip › iTransformer-main/figures/boosting.png]

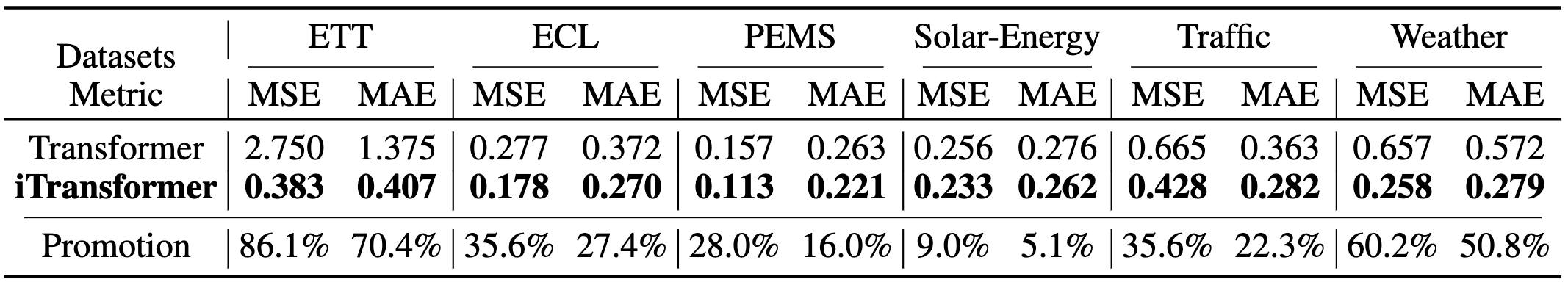

Supplement: S5 Data — (ZIP) [file pone.0319786.s005.zip › iTransformer-main/figures/boosting_trm.png]

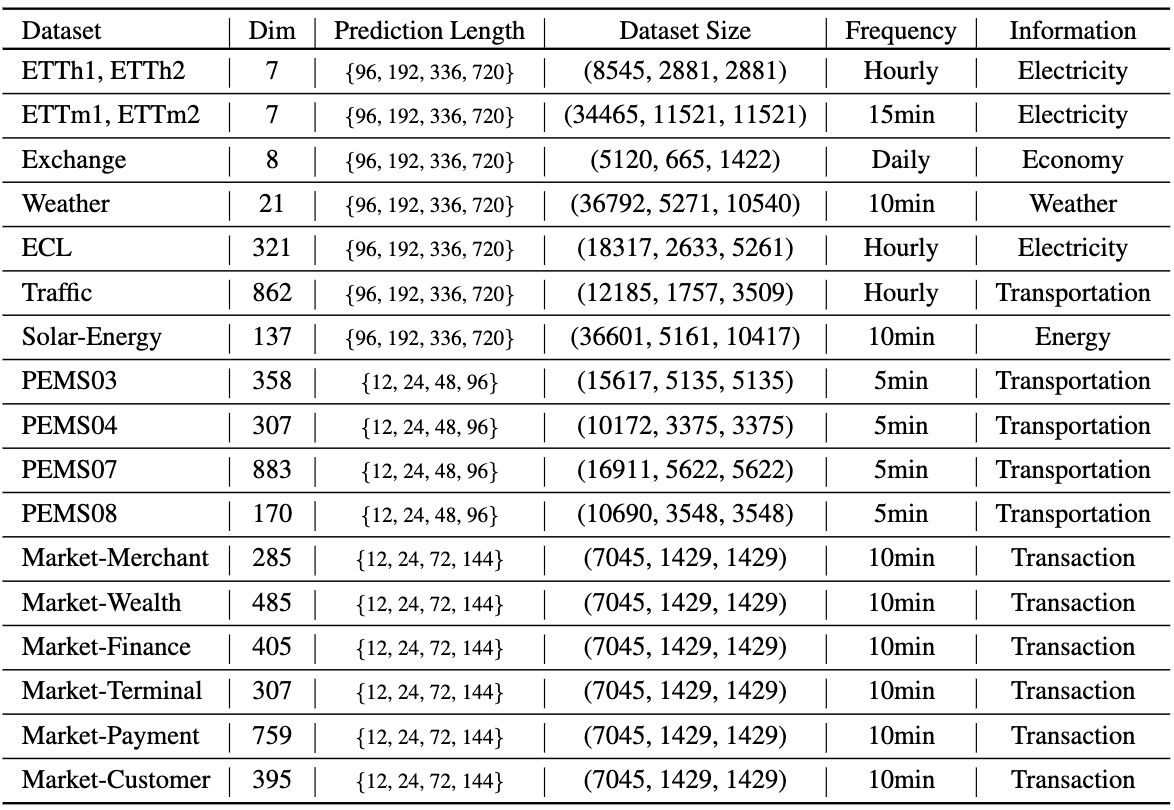

Supplement: S5 Data — (ZIP) [file pone.0319786.s005.zip › iTransformer-main/figures/datasets.png]

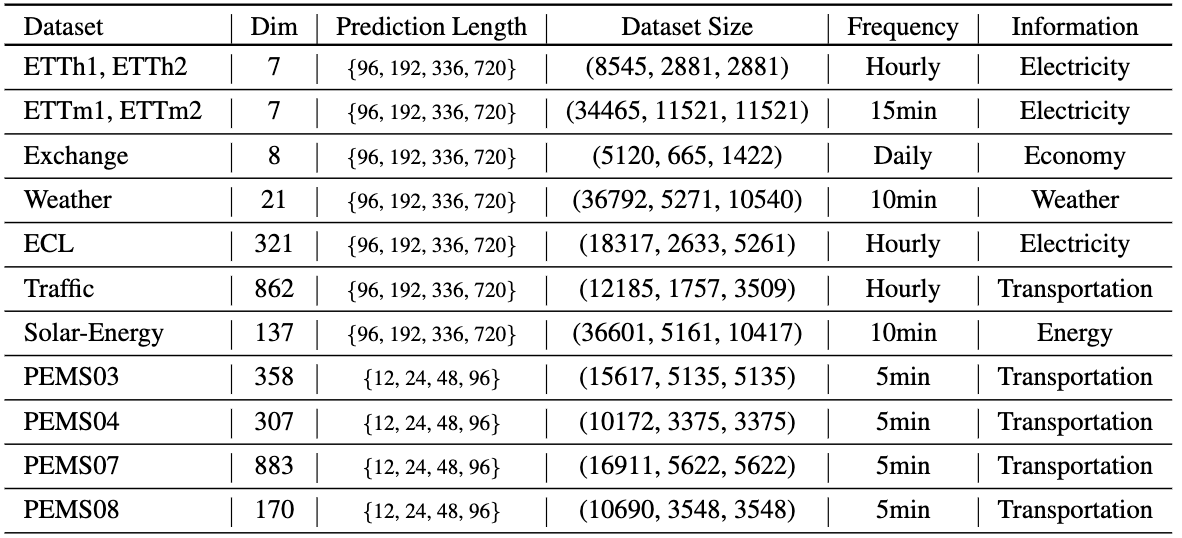

Supplement: S5 Data — (ZIP) [file pone.0319786.s005.zip › iTransformer-main/figures/datasets_mtsf.png]

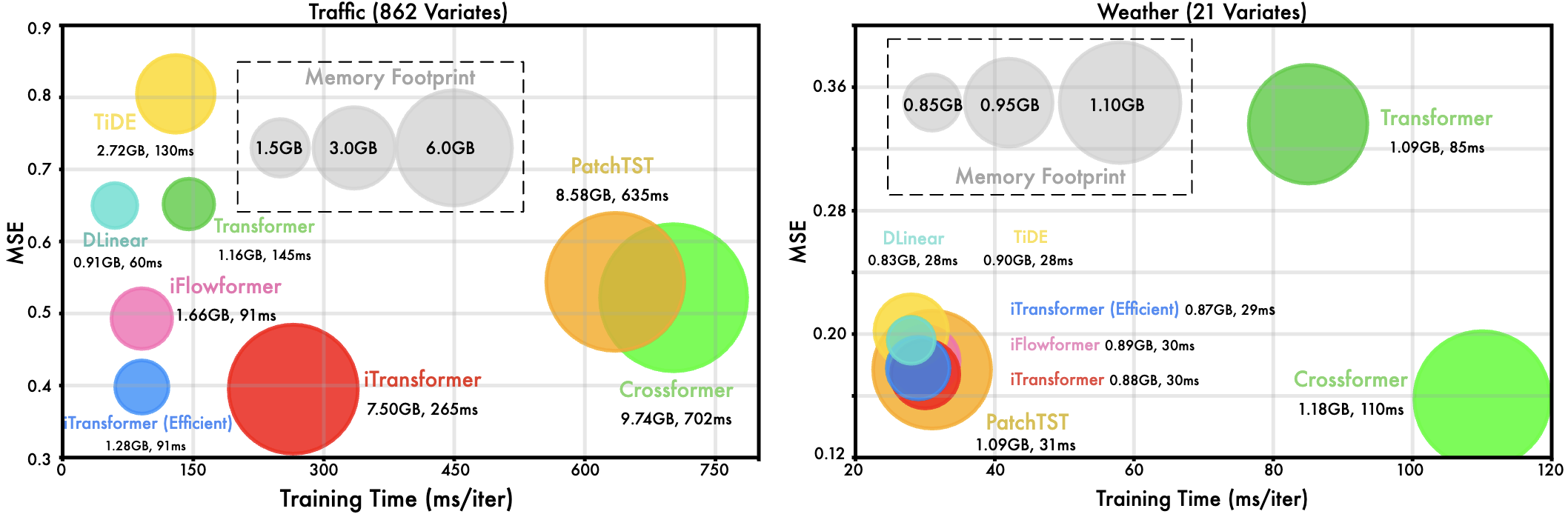

Supplement: S5 Data — (ZIP) [file pone.0319786.s005.zip › iTransformer-main/figures/efficiency.png]

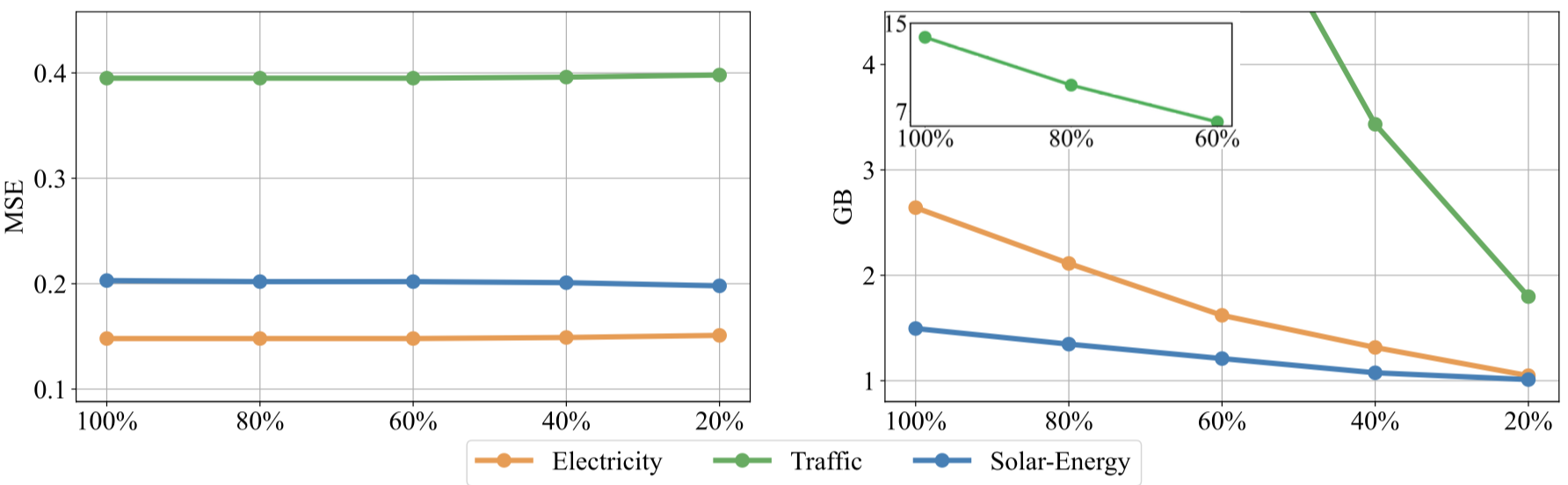

Supplement: S5 Data — (ZIP) [file pone.0319786.s005.zip › iTransformer-main/figures/efficient.png]

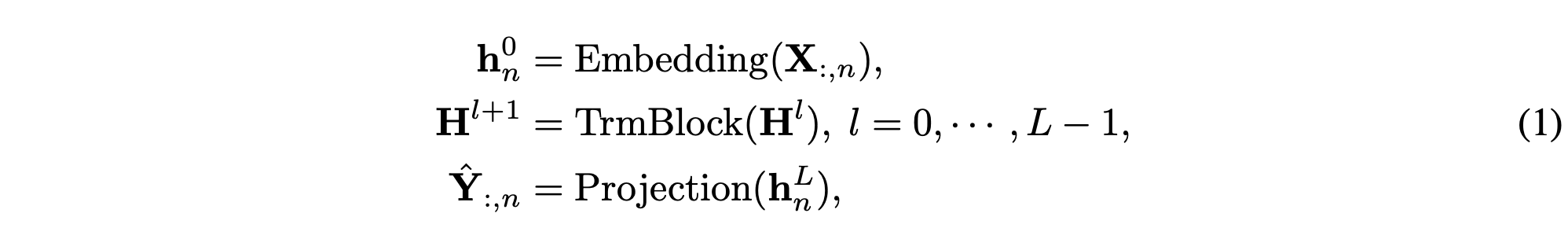

Supplement: S5 Data — (ZIP) [file pone.0319786.s005.zip › iTransformer-main/figures/formulations.png]

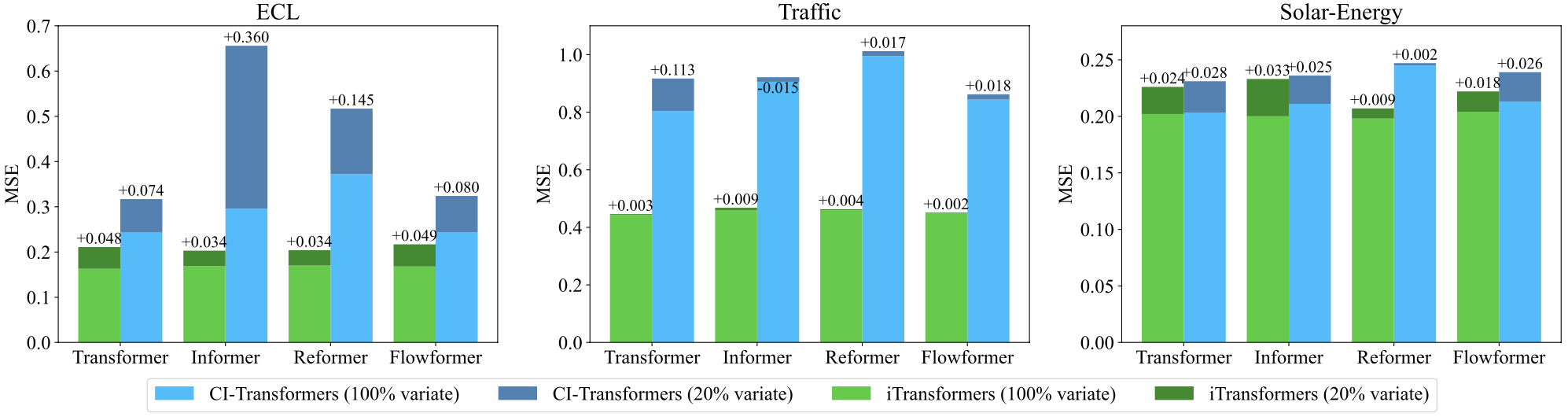

Supplement: S5 Data — (ZIP) [file pone.0319786.s005.zip › iTransformer-main/figures/generability.png]

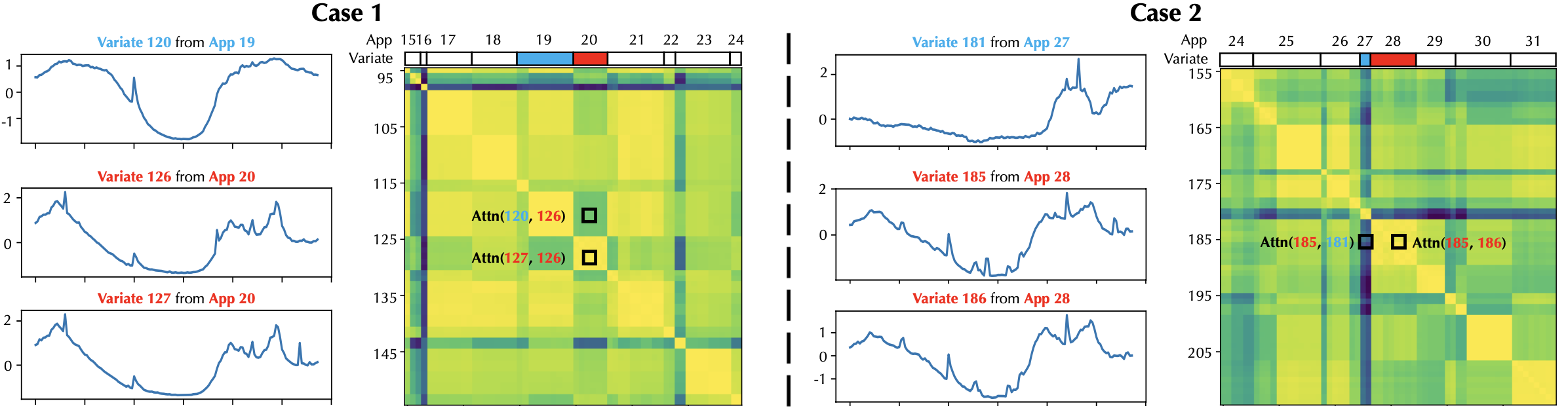

Supplement: S5 Data — (ZIP) [file pone.0319786.s005.zip › iTransformer-main/figures/groups.png]

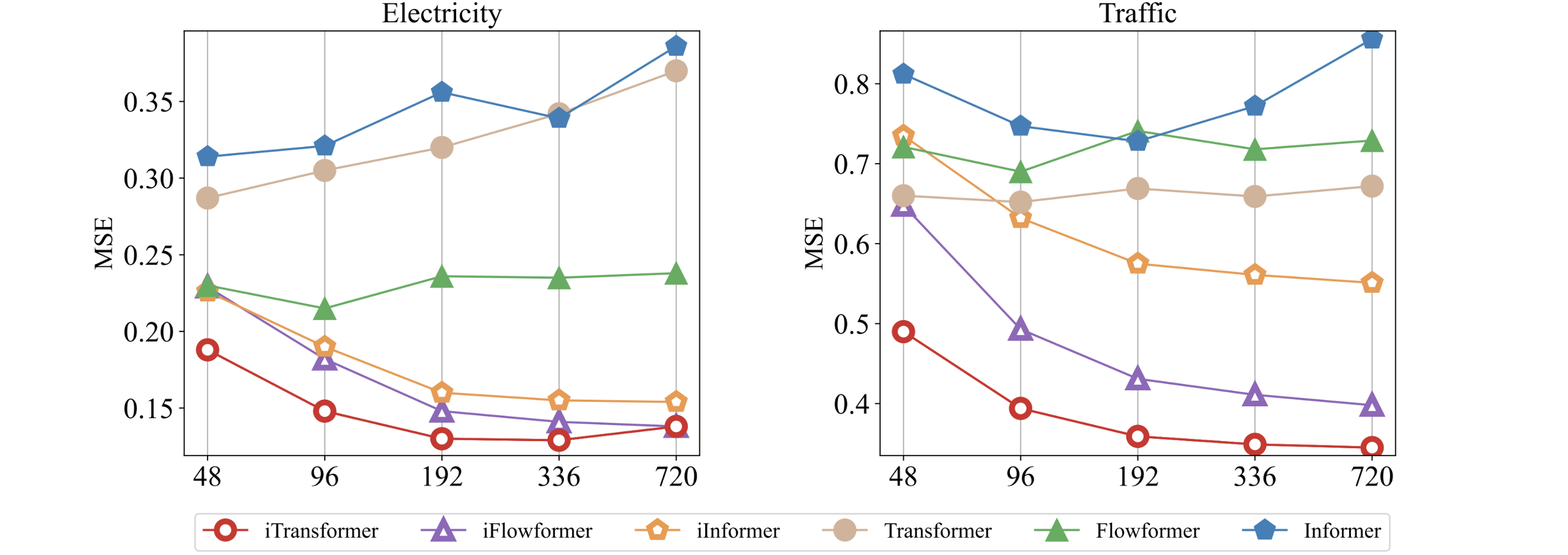

Supplement: S5 Data — (ZIP) [file pone.0319786.s005.zip › iTransformer-main/figures/increase_lookback.png]

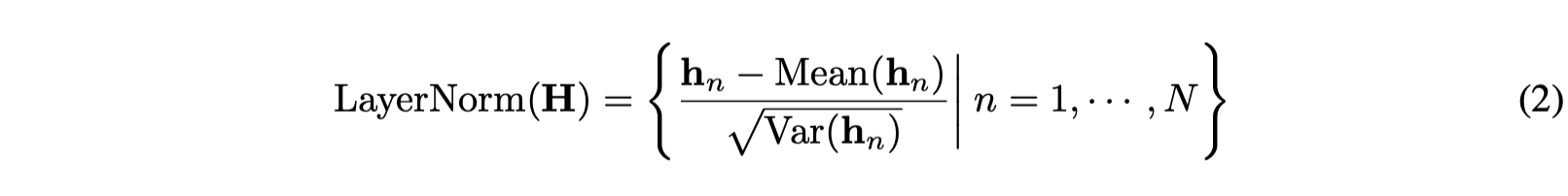

Supplement: S5 Data — (ZIP) [file pone.0319786.s005.zip › iTransformer-main/figures/layernorm.png]

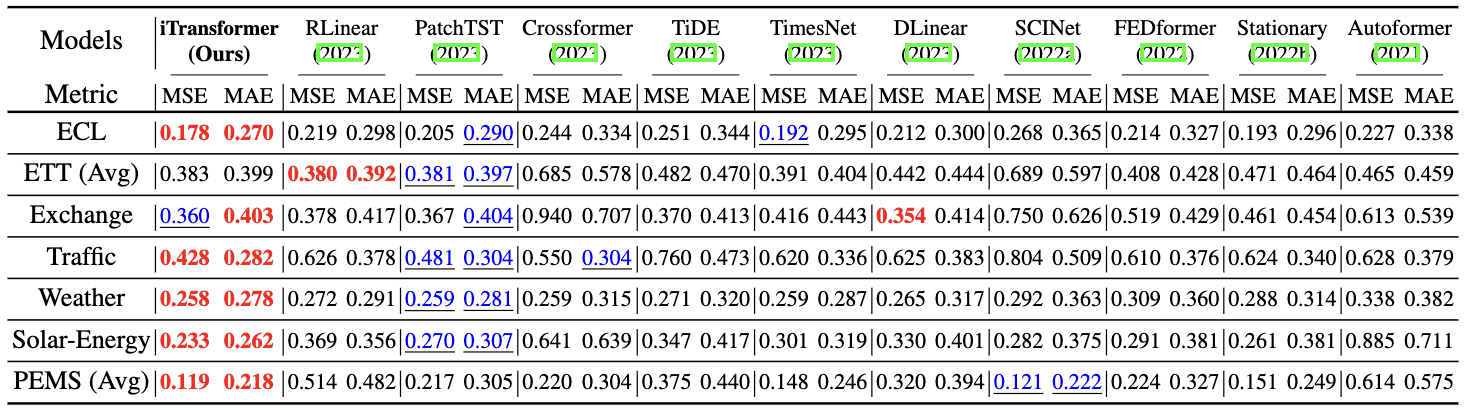

Supplement: S5 Data — (ZIP) [file pone.0319786.s005.zip › iTransformer-main/figures/main_results.png]

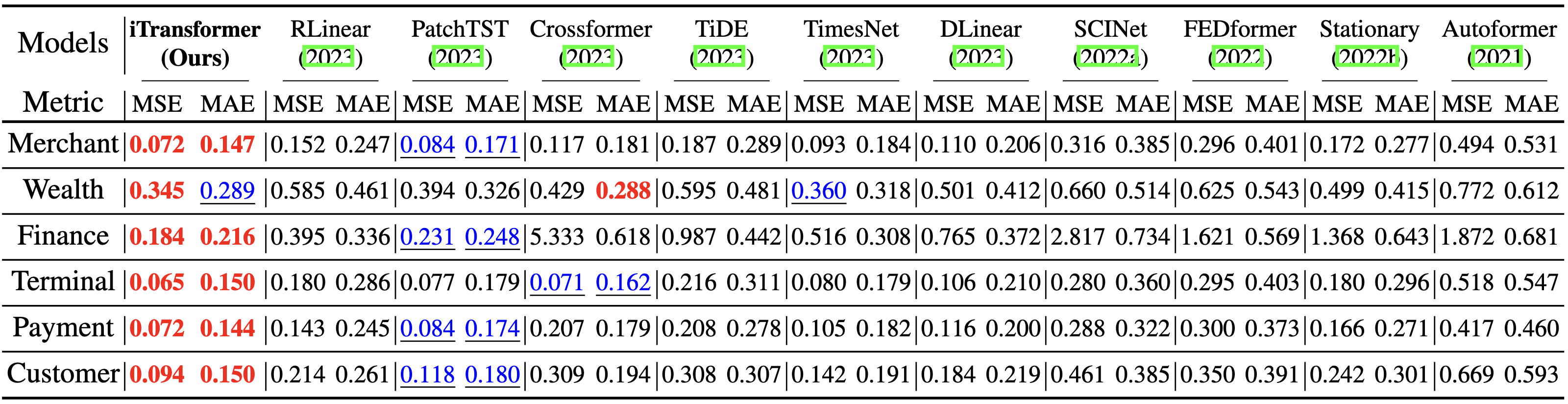

Supplement: S5 Data — (ZIP) [file pone.0319786.s005.zip › iTransformer-main/figures/main_results_alipay.png]

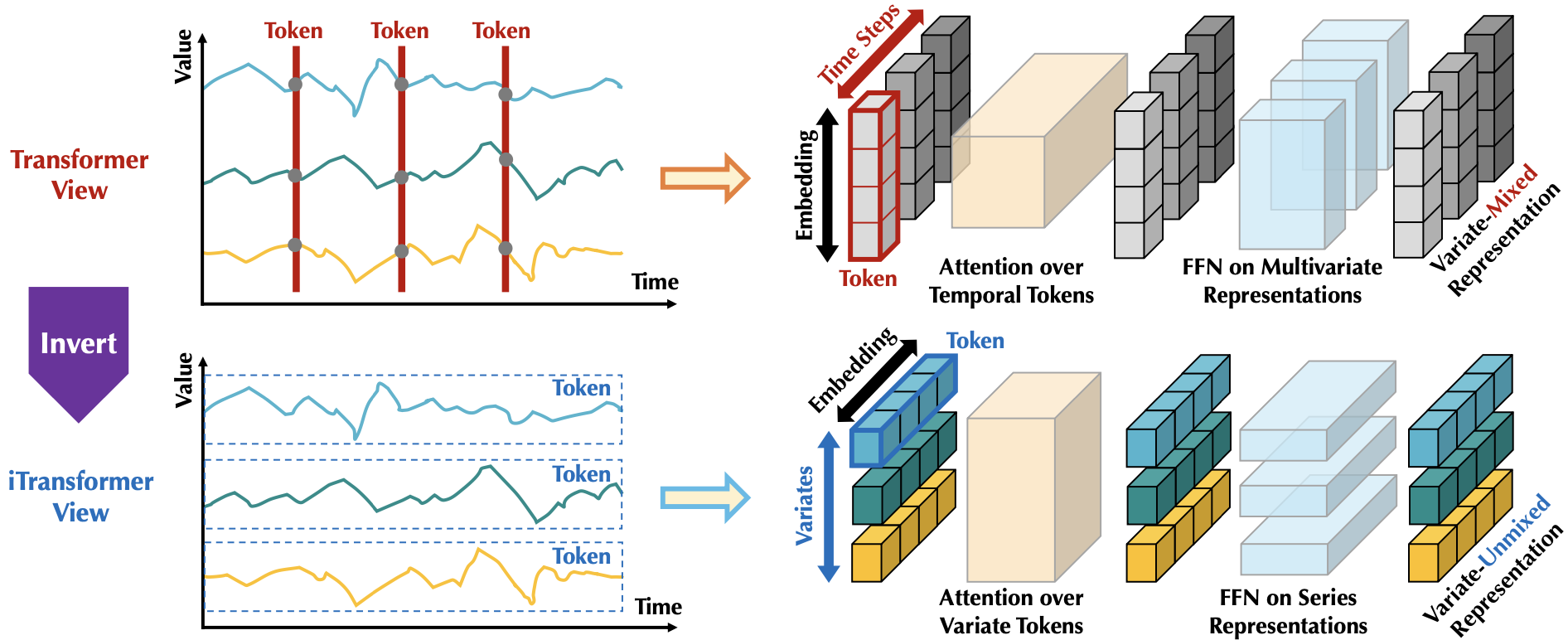

Supplement: S5 Data — (ZIP) [file pone.0319786.s005.zip › iTransformer-main/figures/motivation.png]

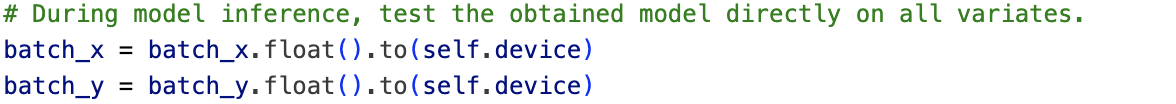

Supplement: S5 Data — (ZIP) [file pone.0319786.s005.zip › iTransformer-main/figures/pi.png]

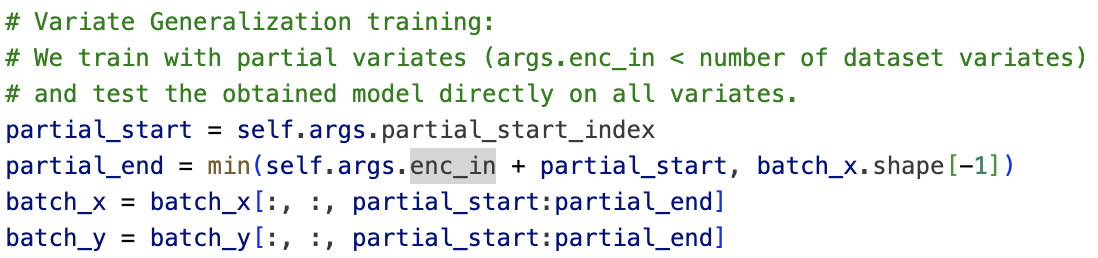

Supplement: S5 Data — (ZIP) [file pone.0319786.s005.zip › iTransformer-main/figures/pt.png]

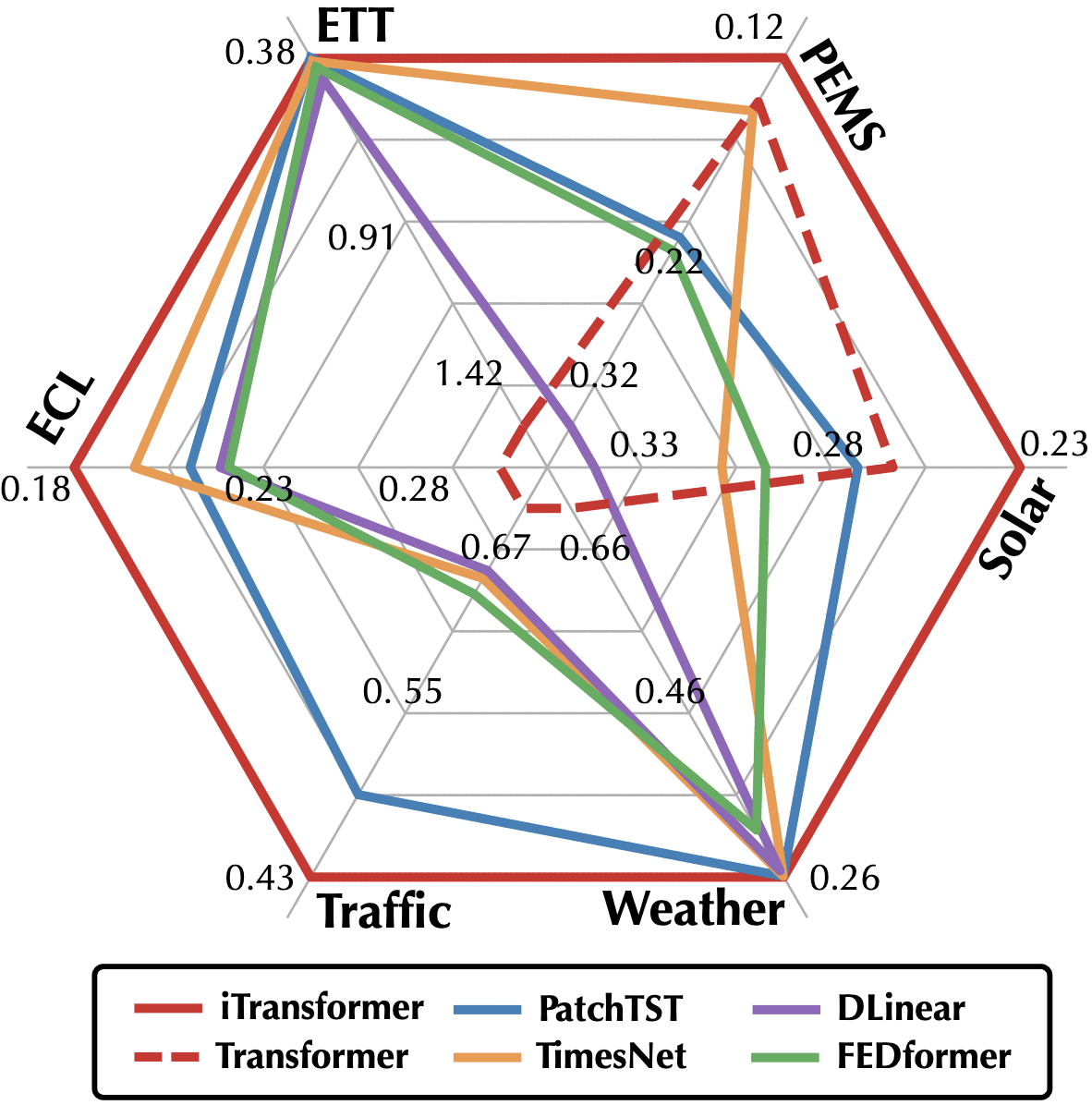

Supplement: S5 Data — (ZIP) [file pone.0319786.s005.zip › iTransformer-main/figures/radar.png]
